# Nanoparticle Thin Films for Gas Sensors Prepared by Matrix Assisted Pulsed Laser Evaporation

**DOI:** 10.3390/s90402682

**Published:** 2009-04-16

**Authors:** Anna Paola Caricato, Armando Luches, Roberto Rella

**Affiliations:** 1 University of Salento, Department of Physics, Via Arnesano, 73100 Lecce, Italy; E-Mail: caricato@le.infn.it;; 2 Istituto per la Microelettronica ed i Microsistemi (IMM) CNR, Via Monteroni, 73100 Lecce, Italy; E-Mail: roberto.rella@le.imm.cnr.it

**Keywords:** Nanoparticles, thin films, MAPLE, gas sensors

## Abstract

The matrix assisted pulsed laser evaporation (MAPLE) technique has been used for the deposition of metal dioxide (TiO_2_, SnO_2_) nanoparticle thin films for gas sensor applications. For this purpose, colloidal metal dioxide nanoparticles were diluted in volatile solvents, the solution was frozen at the liquid nitrogen temperature and irradiated with a pulsed excimer laser. The dioxide nanoparticles were deposited on Si and Al_2_O_3_ substrates. A rather uniform distribution of TiO_2_ nanoparticles with an average size of about 10 nm and of SnO_2_ nanoparticles with an average size of about 3 nm was obtained, as demonstrated by high resolution scanning electron microscopy (SEM-FEG) inspections. Gas-sensing devices based on the resistive transduction mechanism were fabricated by depositing the nanoparticle thin films onto suitable rough alumina substrates equipped with interdigitated electrical contacts and heating elements. Electrical characterization measurements were carried out in controlled environment. The results of the gas-sensing tests towards low concentrations of ethanol and acetone vapors are reported. Typical gas sensor parameters (gas responses, response/recovery time, sensitivity, and low detection limit) towards ethanol and acetone are presented.

## 1. Introduction

The synthesis of nanoparticles of different materials and the study of their properties and possible applications are at present among the most active research areas. Nanostructured films are expected to offer efficient and flexible tools for applications in different fields such as catalysis, information storage, nonlinear optics, optical and chemical sensors and many others. The functional properties of the nanostructured films are largely determined by composition, size, morphology and surface properties of the nanoparticles forming the film. Nanoparticles can have very different properties with respect to the relative bulk material due, for example, to quantum confinement effects [1].

High quality nanoparticle films can be obtained by different methods like molecular beam epitaxy (MBE) [2] and metal-organic chemical vapor deposition (MOCVD) [3]. However, deposition processes are very expensive, long and delicate. Moreover, when fabricated with the above-mentioned techniques, nanoparticles have a size distribution of 10–20 % and more and are usually embedded in semiconductor or dielectric layers, thus preventing their growth on non planar substrates and their mixing with other materials.

Pulsed Laser Deposition (PLD) [4] is a much faster method to fabricate nanoparticles and nanoparticle films directly from bulk targets. Target ablation is performed in a noble gas or nitrogen atmosphere at pressures of the order of 100 Pa with nanosecond pulses [5] or in vacuum with femtosecond pulses [6]. Even if some obtained results are of interest, the problem of the large size distribution poses strong limits to the extensive use of PLD for nanoparticle film fabrication. Recently, nanoparticles with well tailored size and low size dispersion were obtained by chemical growth techniques, which are relatively easy and cheap. These techniques can produce spherical colloidal nanoparticles of different materials [7,8]. Colloidal nanoparticles offer great versatility, as they are grown in solution, and they can be incorporated into polymer and glass matrices and into different photonic structures, including microcavities and photonic crystals [9]. The nanocrystals can also be deposited as thin films by spin coating [10] and drop casting [11]. These deposition methods are quite simple and cheap, but they do not ensure a good control of the deposited film thickness and uniform coverage of the substrate, particularly on large areas.

The difficulty of realization of uniform close-packed nanoparticle thin films is a very strong limit to their possible applications. This is why a versatile, fast deposition technique, like MAPLE, was considered as a very attractive alternative. Colloidal nanosized particles, prepared with the required uniform dimensions, can be diluted in a volatile solvent and frozen at the liquid nitrogen temperature, thus forming the target to be laser irradiated.

Recently, TiO_2_ and SnO_2_ colloidal nanoparticle films were prepared by MAPLE deposition, preserving the size and crystalline phase of the starting particles [12,13]. The interest was in testing nanoparticle films as gas sensors. In fact, the electrical properties of semiconducting dioxides like TiO_2_ and SnO_2_, are influenced by gaseous ambient [14]. The use of nanoparticles increases the sensitivity of the sensor. This improvement is attributed to the higher density of surface sites available for gas adsorption in nanocrystalline materials, as compared to that provided by the corresponding bulk material [15]. A summary of the methodology and of the obtained results [12,13,16,17] is given here.

## 2. Experimental Apparatus and Procedure

**Figure 1.**
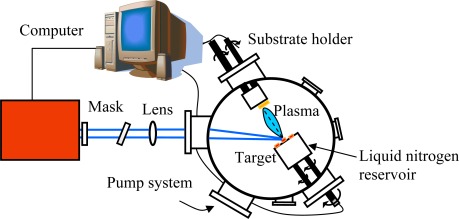
Schematic diagram of a MAPLE deposition system.

The MAPLE deposition hardware does not substantially differ from that commonly used in PLD. Excimer lasers (or Nd:YAG, third harmonic at 335 nm) are mostly used, since UV radiation strongly couples with almost any target material. The main difference with respect to PLD is the target holder, since it must be kept at very low temperature during depositions. It means that a liquid nitrogen reservoir must be connected to the target holder. It is usually made of high-conductivity oxygen-free cooper, crossed by a stem of the same material supporting the target holder. The target must rotate (3–10 Hz), like in PLD, to allow smooth erosion of the frozen solution. Feedthroughs and connectors have to be accurately designed, with properly chosen gaskets, to allow rotation at low temperature without seizing problems. A schematic diagram of a MAPLE deposition system is shown in Figure 1.

Titanium dioxide colloidal nanoparticles (size 10±1 nm) in the anatase phase were prepared by using standard procedures [18]: 1 mL of Ti(IV) tert-butoxide, was added to benzyl alcohol (5 mL). The reaction mixture was transferred into a Pyrex tube and heated in a furnace at 220 °C for 2 days. The resulting milky suspension was centrifuged, the precipitate thoroughly washed in ethanol and dichloromethane, and subsequently dried in air at 60 °C. The nanoparticles were diluted in deionized water with a concentration of 0.2 wt % and put in an ultrasonic bath for 10 minutes to prevent aggregation. After, the solution was gradually immersed in liquid nitrogen (−196 °C) and solidified to form the MAPLE target, which was quickly mounted inside a vacuum chamber on a target holder, cooled with liquid nitrogen to guarantee a low and constant temperature (− 160 °C). The vacuum chamber was evacuated down to 5×10^−4^ Pa and the frozen target was irradiated with an ArF (λ=193 nm) excimer laser with fluence F=550 mJ/cm^2^, pulse duration τ=20 ns and repetition rate of 10 Hz. During the irradiation process, the pressure into the chamber rose to 4×10^−3^ Pa, due to the laser induced solvent evaporation. The nanoparticles were deposited on substrates placed at 36 mm in front of the target, which was rotated at the frequency of 3 Hz, to allow uniform erosion. The number of subsequent laser pulses applied to deposit a single film was 6,500. Before starting depositions, 200 pulses were used to remove the surface layer of frozen water vapor formed on the target, while a shutter screened the substrate.

Tin dioxide nanoparticles were prepared as follows: in a 500 mL three-neck flask equipped with a condenser and a thermometer, 1.25 g of Sn (II) 2-ethylhexanoate ([CH_3_(CH_2_)_3_CH(C_2_H_5_)CO_2_]_2_Sn) were mixed with 10 mL of diphenyl ether (DPE) and hexylamine, with an hexylamine:Sn molar ratio of 1:1. The flask was thoroughly degassed and heated up to 250 °C. When the final temperature was reached, the liquid was further heated for 25 minutes. After cooling the flask, addition of methanol resulted in precipitation of the nanocrystals, which were collected by centrifugation and further purified by dispersion in hexane and subsequent recovering by addition of methanol. While the as-recovered nanocrystals are usually soluble in hexane or toluene, the solubility is lost through the purification process. In this case, the solubility is fully recovered when the nanocrystals are dispersed in hexane and a few drops of trioctylphosphine are added. The dimensions of the resulting SnO_2_ nanoparticles were of 3.6±0.6 nm, as determined by TEM inspection, with one monolayer of trioctylphosphine as capping layer. They presented the tetragonal (cassiterite) crystallographic phase, as reported in [7]. Then, the colloidal nanoparticles were diluted in toluene with concentration of 1 wt %. Before the freezing procedure at the liquid nitrogen temperature, the nanoparticle solution was put in an ultrasonic bath for 10 minutes to prevent aggregation. The solution was then frozen at liquid nitrogen temperature. The frozen target was irradiated with a KrF (λ=248 nm, τ=20 ns) excimer laser at the fluence of 350 mJ/cm^2^. Each film was deposited with 6,000 laser pulses at the repetition rate of 10 Hz. The target, which was rotated at the frequency of 3 Hz to allow uniform erosion, was placed in front of the substrate at a distance of 36 mm.

The dioxide nanoparticle films were deposited on different substrates: silica, <100> Si and interdigitated alumina (Al_2_O_3_) slabs, for the different characterizations. The interdigitated alumina substrates, 2 mm × 2 mm, used for the gas sensing tests, were equipped, on the back face, with a 50 nm Ti/500 nm Pt meander as heater and, on the front face, with similar Ti/Au interdigitated contacts, in order to polarize the sensors and read the electrical current values during the sensing tests.

The deposited films were analyzed by high resolution Scanning Electron Microscopy (SEM), Fourier Transform Infrared spectroscopy (FTIR) and X-ray diffraction (XRD). UV-Vis transmittance and reflectance spectra of the films deposited on silica substrates were recorded, to evaluate the optical properties of the films. In particular, the crystalline phase of the films was investigated by using an INEL CPS 120 diffractometer, the surface morphology by using a JEOL 6500F Field Emission Scanning Electron Microscope, while a Perkin Elmer apparatus with 4 cm^−1^ resolution in transmission mode was used to record the FTIR spectra in the range 500–3200 cm^−1^ for chemical characterizations. For structural analysis, room temperature UV-Vis transmittance and reflectance spectra of the dioxide films deposited on silica substrates were recorded by a Varian Cary 500 UV-Vis-NIR spectrophotometer, equipped with an integrating sphere, at normal light incidence. A clean silica substrate was used as reference to measure the absolute transmittance of the deposited film.

## 3. Results and Discussion

### 3.1. TiO_2_ nanoparticle films

**Figure 2.**
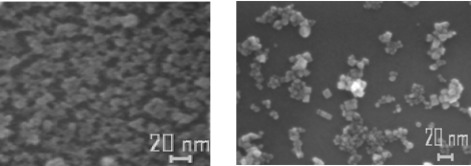
High resolution SEM micrographs of TiO_2_ nanoparticle thin films deposited by MAPLE (left) and spin coating techniques (right) on Si substrates.

**Figure 3.**
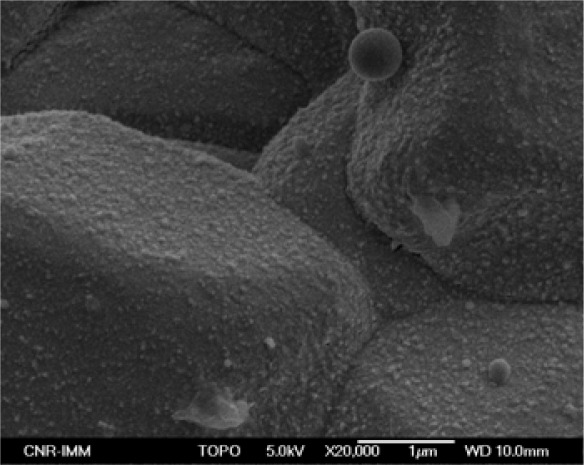
SEM micrographs of TiO_2_ nanoparticle thin film MAPLE-deposited onto a rough alumina substrate.

High resolution SEM images of the TiO_2_ nanoparticle films deposited on silicon substrates showed that the nanoparticles preserved the starting dimensions, although a tendency to form aggregates can be noted. By a comparison with TiO_2_ nanoparticle films deposited by the spin coating technique, starting from a solution with the same TiO_2_ nanoparticle concentration (i.e. 0.2 wt %) used for the MAPLE-deposited films, a much more uniform coverage for the MAPLE deposited film was observed (Figure 2). A very interesting result is that films of nanoparticles following the substrate morphology were deposited also on the rough Al_2_O_3_ substrates used for gas sensing measurements (Figure 3). In fact, rough substrates improve the performance of the sensors by increasing the active area of the sensing films. Moreover, for electrical signal transfer, it is necessary to deposit metal conductors on the insulating substrates. Metal ribbons of course increase the roughness of the substrates. A rather uniform distribution of nanoparticles on rough substrates is essential to get a good electrical response.

**Figure 4.**
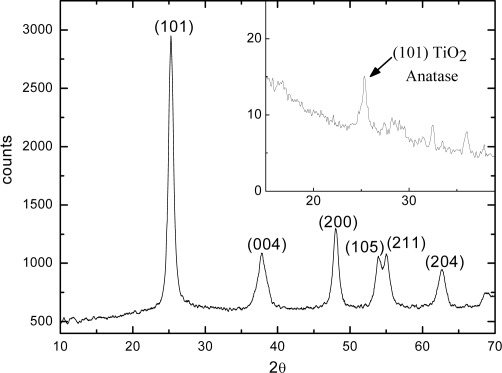
XRD spectra of the TiO_2_ nanocrystals in the starting solution and in the MAPLE-deposited thin film (inset).

The preservation of the anatase crystal phase was evidenced by XRD spectra, where the characteristic peak of the anatase phase at 2θ=25°, corresponding to the reflection by the <101> crystallographic plane, is well evident. In Figure 4 the XRD spectra of the starting solution and of the film deposited on a Si substrate (inset) are shown. Obviously, because of the small thickness of the film (≈ 30 nm, measured performing a scratch on the film and making an AFM measurement transversal to the scratch), the signal coming from the film is lower and noisier with respect to the one of the solution.

The transmittance and the reflectance UV-Vis spectra of TiO_2_ nanoparticle films deposited by the MAPLE technique on silica substrate were acquired in the 200–800 nm spectral range. In general, the transmittance spectrum is characterized by a sharp fall at wavelengths shorter than ∼360 nm, corresponding to the energy threshold for band edge absorption of TiO_2_.

In order to determine the nature of the band gap of the nanostructured material, the spectral behavior near the fundamental absorption edge can be calculated by considering the expression [19]:

(1) $$\alpha h\nu □\;=B{\left(h\nu {-E}_{g}\right)}^{p}$$

where α is the absorption coefficient and E_g_ is the optical energy gap corresponding to the transition indicated by the value of p. In particular, p is 1/2, 3/2, 2 and 3 for direct allowed, direct forbidden, indirect allowed and indirect forbidden transitions, respectively. The factor B depends on the transition probability and can be assumed constant within the investigated optical frequency range. By plotting (αhν)^2^ against the photon energy hν, a linear trend, up to photon energies of about 5 eV, corresponding to allowed direct transition (p=1/2) was obtained. It is well known that anatase TiO_2_ is an indirect gap semiconductor due to the lowest Γ_1_←X_1,2_ transitions. However, absorption thresholds in anatase TiO_2_ nanoparticles were already found at higher energy, with respect to bandgap, and attributed to direct transitions in an otherwise indirect bandgap semiconductor, due to size effects [20, 21]. In our case, the optical energy gap resulted to be of about 3.6 eV, as compared to the bulk anatase TiO_2_ value of 3.18 eV, and close to the value reported in literature for anatase TiO_2_ nanoparticle Langmuir-Blodgett films [21].

### 3.2. SnO_2_ nanoparticle films

**Figure 5.**
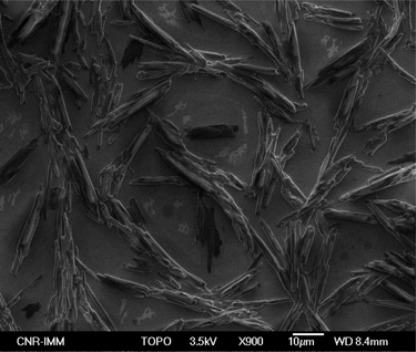
SEM micrograph of the as deposited SnO_2_ film on <100> Si substrate

The first step in the analysis of the deposited films was the SEM characterization, in order to evaluate the film morphology. As shown in Figure 5, the surface of the as-deposited film consists of uniformly distributed elongated structures.

These structures are due to the organic material, trioctylphosphine, present in the solution, as confirmed by their gradual modification during SEM inspection, down to a complete vanishing due to the interaction with the electron beam. Due to the presence of such structures, the analysis of the nanoparticle was not possible. For this reason, the deposited films were annealed in order to remove any residual organic material from the surface of the films.

**Figure 6.**
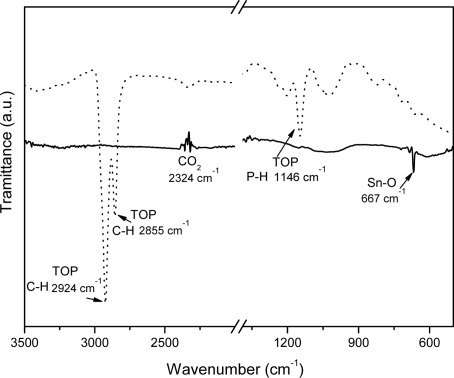
FTIR spectra of the as-deposited (dotted line) and vacuum-annealed (solid line) SnO_2_ nanoparticle films.

To get more information about the film structure and composition before annealing, the FTIR spectrum was acquired (Figure 6, dotted line). Different absorption bands are observed, which can be ascribed predominantly to the trioctylphosphine present into the matrix and in the nanoparticle capping layer. In fact the strong absorption peaks at 2,980-2,850 cm^−1^ are assigned to methyl C-H stretching vibration, while the band at 1,145 cm^−1^ arises from P=O stretching [22]. The bands located around 825 cm^−1^ and 720 cm^−1^ are plausibly ascribed to the vibrational modes of the trioctylphosphine molecules, while the weak band at 667 cm^−1^ is ascribed to the O-Sn-O bond [23]. The band at 2,324 cm^−1^ is due to the atmospheric CO_2_ present in the environment.

The presence of trioctylphosphine was confirmed also by the UV-Vis absorption spectrum (not reported here), recorded in the spectral range of 200–800 nm, which was very similar to the absorption spectrum of the capping material diluted in hexane (the hexane absorption in the investigated spectral range is negligible). On the other hand, the use of trioctylphosphine is necessary to avoid nanoparticle precipitation. However its use determines, as a consequence, the presence of this agent also in the toluene solution with a concentration which can be estimated to be of 10 % in volume. The trioctylphosphine has a vapor pressure of 120 Pa at 20 °C, which is much lower than that of the toluene solvent (2.9×10^3^ Pa). As a consequence, it is not effectively pumped out during the MAPLE deposition process and finally a trioctylphosphine layer is deposited on the substrate, contributing to the composition of the films.

**Figure 7.**
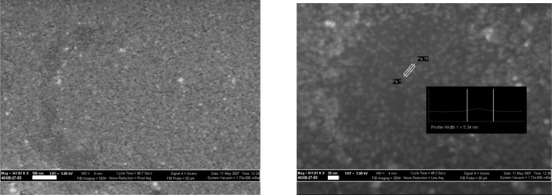
Left: overview of the annealed SnO_2_ nanoparticle film morphology (the white marker is 100 nm). Right: a higher magnification zone of the film (the white marker is 20 nm).

In order to remove the trioctylphosphine layer, the deposited films were annealed at 400 °C either in vacuum (∼4–5×10^−3^ Pa) or in dry air for 1 h. Similar results were obtained in both cases. The SEM micrograph of one vacuum-annealed film deposited on <100> Si is shown in Figure 7.

No trace of the trioctylphosphine layer was detected on the annealed film, which is constituted by a rather uniform distribution of nanoparticles. Occasionally, circular shaped areas are apparent on the film, formed by a less dense nanoparticle distribution. From higher magnification SEM figures, it was possible to measure the dimensions of the single nanoparticles. The average nanoparticle dimension resulted to be of 4±1 nm, in accordance with the nanoparticle dimension of the starting solution (3.6±0.6 nm). Some bigger nanoparticles, with dimensions of 10–20 nm, were also evidenced, probably due to nanoparticle coalescence caused by the post-deposition annealing or to the presence of agglomerates in the starting solution itself. The SEM analysis also allowed estimating a film thickness of about 7±3 nm.

The FTIR analysis was repeated on the annealed films and the resulting spectrum can be seen in Figure 6 (solid line). All the bands ascribed to trioctylphosphine or toluene are not present anymore. Only one peak is clearly visible at 667 cm^−1^, attributed to the vibration of the antisymmetric O-Sn-O bridging bond [23]. This spectrum confirms the solvent and capping elimination after annealing at moderate temperature and the formation of a film composed of SnO_2_ nanoparticles, only.

**Figure 8.**
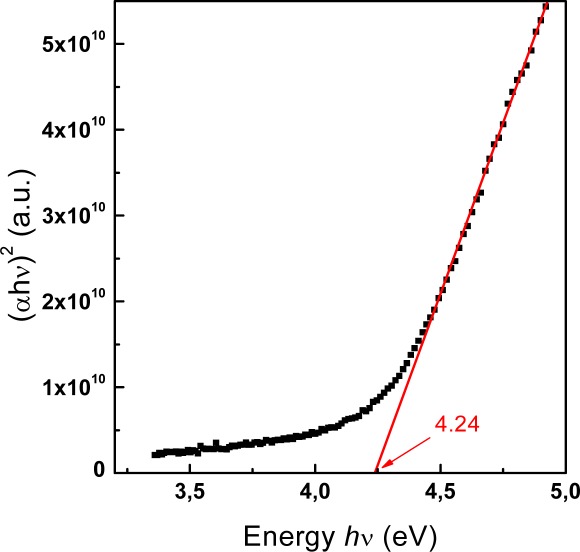
Plot of (αhν)^2^ vs photon energy hν of the SnO_2_ nanoparticle film (open dotted line). The closed line represents the linear fit.

UV-visible absorption spectra were recorded to characterize the optical absorbance of the nanocrystalline SnO_2_ films. The optical energy gap *E_g_* can be determined by using the well known equation (1). Figure 8 shows the plot of (*αhν*)^2^ versus photon energy hν for the SnO_2_ nanocrystalline film deposited on a silica substrate, indicating a direct allowed transition for the film. The absorption coefficient α was determined directly from the spectrophotometer readings of the SnO_2_ film/substrate and of the bare glass substrate. For the SnO_2_ film an average thickness of two monolayers (∼ 7.2 nm), as evaluated from the SEM analyses, was assumed for computations. From the linear fitting and the linear extrapolation to the zero ordinate (αhν)^2^=0 an energy gap value of 4.24 eV was obtained. This value is higher than that one of 3.6 eV reported in literature for bulk SnO_2_ [24]. The observed blue shift of the absorption edge is due to the small dimension of the nanocrystalline particles. In fact, the optical band gap of the nanocrystalline particles depends on the particle radius, due to quantum confinement of electrons and holes, as reported by different authors [25,26].

**Figure 9.**
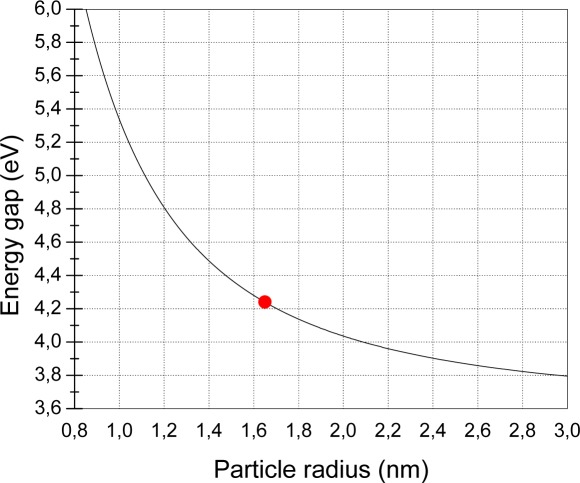
Calculated SnO_2_ nanoparticle energy gap vs. particle radius. The dot represents our sample characteristics (E_g_=4.24 eV).

The dependence of absorption onset on the particle size is based on the effective mass approximation and the increase in the optical band gap of a nanocrystalline semiconductor may be described by the relation [27]:

(2) $$E^{*}=E_{g}+\frac{h^{2}}{8R^{2}}\left(\frac{1}{m_{e}}+\frac{1}{m_{h}}\right)-\frac{1.8e^{2}}{\varepsilon R}$$

where E^*^ and *E_g_* are the cluster and bulk-state band gap energies, respectively; *m_e_* and *m_h_* are the electron and hole effective mass, respectively; *ε* is the dielectric constant of the semiconductor and *R* is the average particle size, while *h* is the Plank constant and *e* is the electron charge. Generally for SnO_2_ we can put *E_g_*=3.6 eV, *ε*=12, *m_e_*=0.3 *m_0_* and *m_h_*=0.8 *m_0_*, where *m_0_* is the free electron mass [28]. The above relation is plotted in Figure 9. From this figure, in correspondence to an energy gap of 4.24 eV as deduced from the absorption spectrum, an average particle diameter of 3.3 nm can be determined. This value is in good agreement with the one inferred from SEM analysis and with the starting particle dimension. The slightly lower value obtained from the optical measurements can be attributed to a probable contribution of the defect states present on the nanoparticle surface, due to the removal of the capping layer during the annealing process.

## 4. Sensor fabrication and characterization

**Figure 10.**
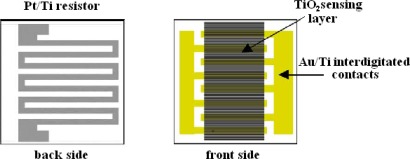
Back and front side of the sensing element.

**Figure 11.**
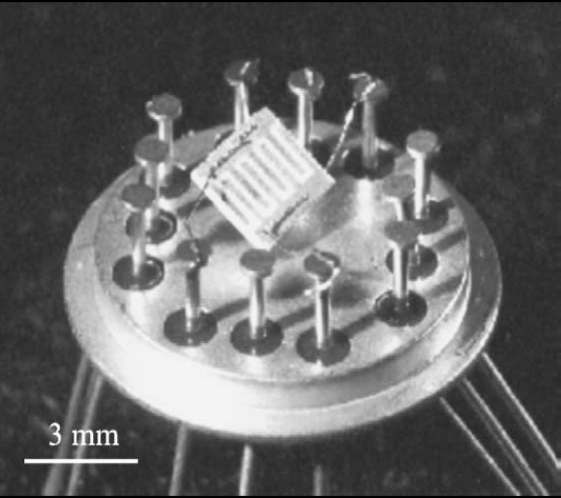
Sensing elements fixed on wired socket.

To realize a sensor based on the electrical transduction mechanism, nanoparticle dioxide layers were MAPLE deposited on pre-processed alumina substrates. The alumina substrates (2 mm × 2 mm) were patterned, on the back face, with 50 nm Ti/500 nm Pt meander as heater and, on the front face, with similar Au/Ti interdigitated contacts, in order to read the electrical current values during tests (Figure 10). Each substrate was then soldered onto a commercial TO-8 socket (Figure 11) and hosted in a suitable test chamber.

Gas test measurements were carried out in the constant temperature mode by recording the dynamic changes of the electrical resistance caused by the exposition to different concentrations of ethanol and acetone vapors, since surface reactions with reducing chemical species as ethanol and acetone cause an increase in electrical conductance.

**Figure 12.**
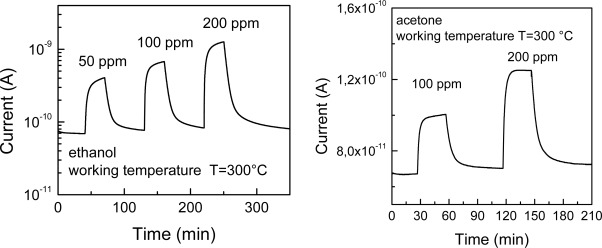
Typical dynamic response of the MAPLE deposited TiO_2_ sensing layer for different concentrations of ethanol (right) and acetone (left) vapors at the working temperature of 300 °C.

For TiO_2_-based sensors the ethanol and acetone vapor concentrations were varied from 20 to 200 ppm in dry air. The sensor working temperature was varied from 250 to 500 °C to find the best operating temperature. As an example, Figure 12 shows typical dynamic changes in the electrical current of a MAPLE deposited TiO_2_ nanoparticle film, at the working temperature of 300 °C, for ethanol vapors at different concentrations, using a working voltage of 10 V.

**Figure 13.**
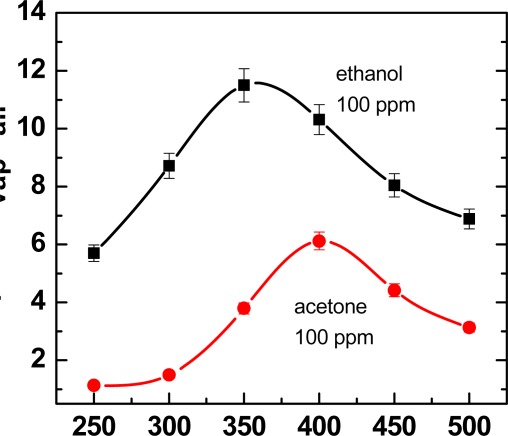
Response of the TiO_2_-based sensor to ethanol and acetone as a function of working temperature.

The relative variation of signal in electrical current is very high (up to about 1 order of magnitude) even at very low concentrations of both the considered vapors. These very good gas-sensing properties towards ethanol and acetone may be attributed to the nanoscale dimensions of the TiO_2_ particles. The measurements highlight also the stability of the electrical current signal of the MAPLE-deposited TiO2 nanoparticle thin film. Moreover, also the signal recovery is complete when the air flux is restored after the gas test. It can be also noticed that the response time is not very fast (∼ 4 minutes). We found that it is influenced by the working temperature. In fact, it has been shown that the working temperature has a strong influence on the gas response and on the dynamic behavior of the responses. The best sensitivities were measured for temperatures of 350 °C and 400 °C for ethanol and acetone vapors, respectively (Figure 13). The calibration curves showed a higher sensitivity of the TiO_2_ nanoparticle film to ethanol with respect to acetone.

**Figure 14.**
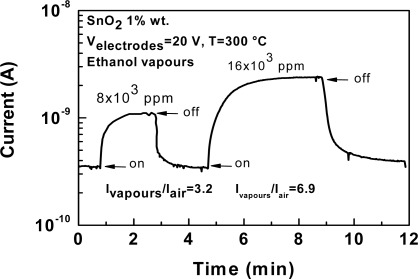
Typical dynamic response of the MAPLE deposited SnO_2_ sensing layer for concentrations of ethanol vapors of 8 and 16×10^3^ ppm at the working temperature of 300 °C.

SnO_2_ nanoparticle films were also deposited on interdigitated alumina substrates, following the same procedure. After deposition, the devices were soldered onto TO-8 sockets and hosted in a suitable test chamber. First, gas sensing tests were carried out on the as-deposited MAPLE SnO_2_ films. As expected, no response towards low concentration of ethanol (200 ppm in dry air) was observed, due to the presence of trioctylphosphine. When the sensor working temperature reached 300 °C, a very low variation of the sensor signal was detected at high ethanol concentrations (Figure 14).

**Figure 15.**
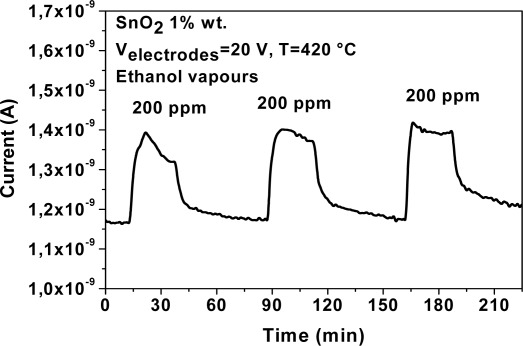
Typical dynamic response of the MAPLE deposited SnO_2_ sensing layer after 1 h annealing in air at 400 °C for concentrations of ethanol vapors of 200 ppm at the working temperature of 420 °C.

An improvement of the electrical properties of the MAPLE deposited SnO_2_ nanoparticle gas sensors were obtained after annealing at 400 °C in air (Figure 15). This can probably be due to a complete removal of trioctylphosphine and to better electrical connections among the nanoparticles.

The sensor showed a high resistance (∼10^10^–10^11^ Ω). This behavior can be linked to the very small SnO_2_ nanoparticle size (∼3.6 nm), to the low thickness of the film (about two monolayers) and to the electrical transport properties through the network of nanoparticles. It is generally agreed that the gas sensing property of the nanoparticle film enhances with decreasing of the grain size in metal oxide films [29]. But, a critical limit of a few nanometers should obviously exist. It must be noted that the understanding of the gas detection mechanism and the transduction function of the gas sensing layer is complex and no comprehensive theory related to the electrical and gas sensing properties of such nanostructured materials is reported in literature.

## 5. Conclusions

It has been demonstrated that MAPLE is a very promising technique for the deposition of nanoparticle films, starting from colloidal solutions. A uniform layer of TiO_2_ nanoparticles was obtained, not only on flat SiO_2_ substrates, but even on rough alumina substrates. Nanoparticle TiO_2_ films were found very sensitive to ethanol and acetone vapors. In contrast, SnO_2_ nanoparticle films were obtained after annealing of the as-deposited layer at the temperature of 400 °C. The annealing procedure was necessary to remove the trioctylphosphine present in the colloidal solution. Sensing characteristics of SnO_2_ nanoparticle films resulted poor, with respect to the ones of TiO_2_ nanoparticle films, most probably due to the very small nanoparticle dimensions (3.6 nm, compared to 10 nm for TiO_2_).
